# Identification of key genes in human airway epithelial cells in response to respiratory pathogens using microarray analysis

**DOI:** 10.1186/s12866-018-1187-7

**Published:** 2018-06-08

**Authors:** Yinghua Li, Guangnan Liu, Jianquan Zhang, Xiaoning Zhong, Zhiyi He

**Affiliations:** 1 Department of Respiratory Medicine, the First Affiliated Hospital of Guangxi Medical University, Nanning, 530021 Guangxi China; 2 Department of Respiratory Medicine, the Second Affiliated Hospital of Guangxi Medical University, Nanning, 530007 Guangxi China

**Keywords:** Microarray analysis, Bioinformatics analysis, Respiratory pathogen, Human airway epithelial cell, Biomarker

## Abstract

**Background:**

Airway epithelium is the primary target for pathogens. It functions not only as a mechanical barrier, but also as an important sentinel of the innate immune system. However, the interactions and processes between host airway epithelium and pathogens are not fully understood.

**Results:**

In this study, we identified responses of the human airway epithelium cells to respiratory pathogen infection. We retrieved three mRNA expression microarray datasets from the Gene Expression Omnibus database, and identified 116 differentially expressed genes common to all three datasets. Gene functional annotations were performed using Gene Ontology and pathway analyses. Using protein-protein interaction network analysis and text mining, we identified a subset of genes functioned as a group and associated with infection, inflammation, tissue adhesion, and receptor internalization in infected epithelial cells. These genes were further identified in BESE-2B cells in response to *Talaromyces marneffei* by Real-Time quantitative PCR (qRT-PCR). In addition, we performed an in silico prediction of microRNA-target interactions and examined our findings.

**Conclusions:**

Using bioinformatics analysis, we identified several genes that may serve as biomarkers for the diagnosis or the surveillance of early respiratory tract infection, and identified additional genes and miRNAs that warrant further fundamental experimental research.

**Electronic supplementary material:**

The online version of this article (10.1186/s12866-018-1187-7) contains supplementary material, which is available to authorized users.

## Background

Respiratory tract infections are common diseases caused by a number of diverse pathogens. These diseases are serious public health concerns globally and pose significant challenges for the World Health Organization. Although treatment using potent anti-infection drugs and effective vaccines has greatly reduced mortality worldwide, respiratory tract infections remain a leading cause of death, in particular, for developing countries [1]. In 2015, there were 17.2 billion cases of upper respiratory infections [2]. A respiratory tract infection is a major risk to patients with chronic respiratory diseases, such as asthma, chronic obstructive pulmonary diseases, and bronchiectasis. Infections caused by viral and/or bacterial pathogens exacerbate chronic respiratory diseases [3].

The airway epithelium is an extremely important barrier against respiratory pathogens. It covers a large surface area and is the primary target for respiratory pathogens [4]. In addition to acting as a natural barrier, recent studies found that airway epithelium functions as an important sentinel of the innate immune system against pathogens [5]. Interactions between epithelial cells and pathogens are inherently complex and encompass many different factors, including adhesion, internalization, and induction of the immune response [6]. In addition to being able to invade epithelial cells, some pathogens are capable of reproducing within infected cells, and in avoiding detection by the host immune system [7]. Therefore, prevention of pathogens from invading airway epithelial cells is crucial for the prophylaxis and treatment of respiratory infections. In the present study, we investigated interactions between airway epithelium cells and respiratory pathogens to better understand the underlying pathogenesis. Our findings may provide improved prophylaxis, surveillance, earlier accurate diagnosis, and better treatments.

Currently, high-throughput technologies such as microarrays and next-generation sequencing combined with bioinformatics analysis enable the generation and analysis of very large datasets, including mRNA, miRNA, and long non-coding RNA expression profiles, and DNA methylation. Such datasets are available in public archives such as the Gene Expression Omnibus (GEO). In this study, we retrieved three datasets of mRNA expression microarrays from GEO, and using bioinformatics analysis, we identified a group of genes as biomarkers in airway epithelial cells response to infection by respiratory pathogens, and we also identified several candidate targets for further fundamental experimental research.

## Results

### Microarray datasets

The mRNA expression profile datasets GSE3397 (Additional file 1), GSE6802 (Additional file 2), and GSE48466 (Additional file 3) were generated using the GPL570, GPL571 and GPL570 microarray platform at Duke University Medical Center, the Technical University Munich, and the University of Louisville, respectively. Thirty-five samples were used consisting of 10 samples of normal human bronchial epithelial cells and 25 samples of human bronchial epithelial cells exposed to the H1N1 influenza virus, the respiratory syncytial virus, *Staphylococcus aureus*, and *Pseudomonas aeruginosa* (Table 1).

**Table 1:** Microarray datasets of mRNA expression profiles

Dataset	Organization	Platform	Sample (n)		Pathogen	time point (hours)
			Normal	Treated		
GSE3397	Duke University Medical Center	GPL570	4	8	Respiratory syncytial virus(Long strain/lot 15D) [16]	4 and 24
GSE6802	Technical University Munich	GPL571	3	8	Respiratory syncytial virusstrain A2,*Staphylococcus aureus*(ATCC 29213), and Pseudomonas aeruginosa(ATCC 27853) [17]	4
GSE48466	University of Louisville	GPL570	3	9	H1N1 influenza virus(A/Kentucky/180/2010, A/Kentucky/136/2009, and A/Brisbane/59/2007) [18]	36

### Differentially expressed genes

**Fig. 1:**
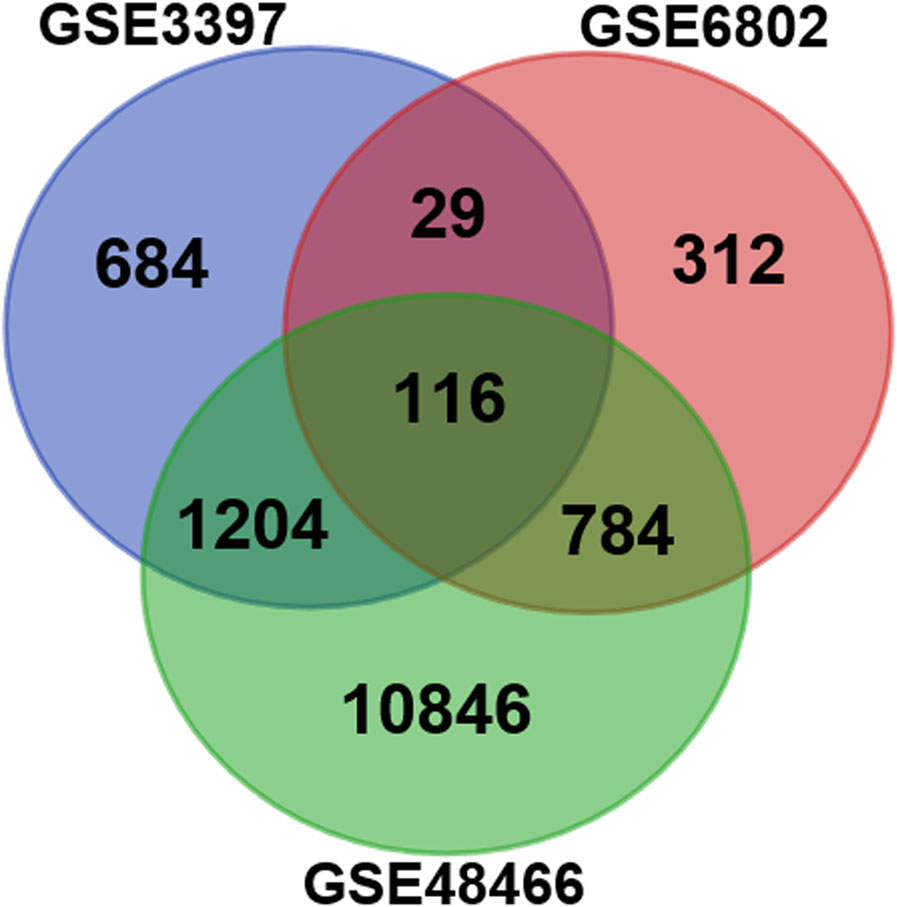
DEGs in three mRNA microarray datasets identified using GEO2R (*P* <  0.05). DEGs between normal and infected airway epithelial cells from the GSE3397 (*n* = 2033), GSE6802 (*n* = 1241), and GSE48466 (*n* = 12,950) datasets were identified, and 116 genes were differentially expressed in all three datasets

Using GEO2R, we identified 2033, 1241, and 12,950 differentially expressed genes (DEGs) between normal and infected airway epithelial cells from the GSE3397, GSE6802, and GSE48466 datasets, respectively. We found that 116 genes were differentially expressed in all three datasets (Fig. 1). This set of 116 genes underwent further evaluation.

### Functional annotations

This subset of 116 genes was uploaded to DAVID database for biological and functional assessments, and Kyoto Encyclopedia of Genes and Genomes (KEGG) pathway analysis. Using Gene Ontology (GO) analysis, we found that these DEGs were usually involved in one of the following 10 biological processes: regulation of foam cell differentiation, regulation of tyrosine phosphorylation of the STAT protein, myeloid leukocyte differentiation, regulation of the JAK-STAT cascade, response to cytokine stimulus, myeloid cell differentiation, cAMP-mediated signaling, anti-apoptosis, ectoderm development and tissue morphogenesis (Table 2). We found that the most significantly enriched pathways were the ubiquitin mediated proteolytic pathway, NOD-like receptor (NLR) signaling pathway, and the apoptosis pathway (Table 3).

**Table 2:** Top 10 enrichment biological processes of the 116 DEGs

ID	Function	Gene	*P*-value
GO:0010743	regulation of foam cell differentiation	CSF2, CSF1, NFKBIA	0.01
GO:0042509	regulation of tyrosine phosphorylation of STAT protein	CSF2, NF2, IL6ST	0.02
GO:0002573	myeloid leukocyte differentiation	CSF2, CSF1, CASP8	0.02
GO:0046425	regulation of JAK-STAT cascade	CSF2, NF2, IL6ST	0.02
GO:0034097	response to cytokine stimulus	IL6ST, CASP8, PML, SERPINA1	0.01
GO:0030099	myeloid cell differentiation	CSF2, CSF1, CASP8, PML	0.02
GO:0019933	cAMP-mediated signaling	OPRM1, EIF4EBP2, PTGER3, NPR3	0.02
GO:0006916	anti-apoptosis	CSF2, SQSTM1, TNFRSF10D, TNFAIP8, SKP2, NFKBIA, TNFAIP3, BIRC3	< 0.01
GO:0007398	ectoderm development	TXNIP, NF2, FOXE1, TFAP2A, EMP1, KLF4	< 0.01
GO:0048729	tissue morphogenesis	NF2, CASP8, FOXE1, TFAP2A, KLF4	0.02

**Table 3:** Pathway enrichment of the 116 DEGs

ID	Definition	Gene	*P*-value
hsa04120	Ubiquitin mediated proteolysis	CUL3, SKP2, PML, NEDD4L, RCHY1, BIRC3	< 0.01
hsa04621	NOD-like receptor signaling pathway	CASP8, NFKBIA, TNFAIP3, BIRC3	0.01
hsa04210	Apoptosis	TNFRSF10D, CASP8, NFKBIA, BIRC3	0.03

### Protein and protein network

**Fig. 2:**
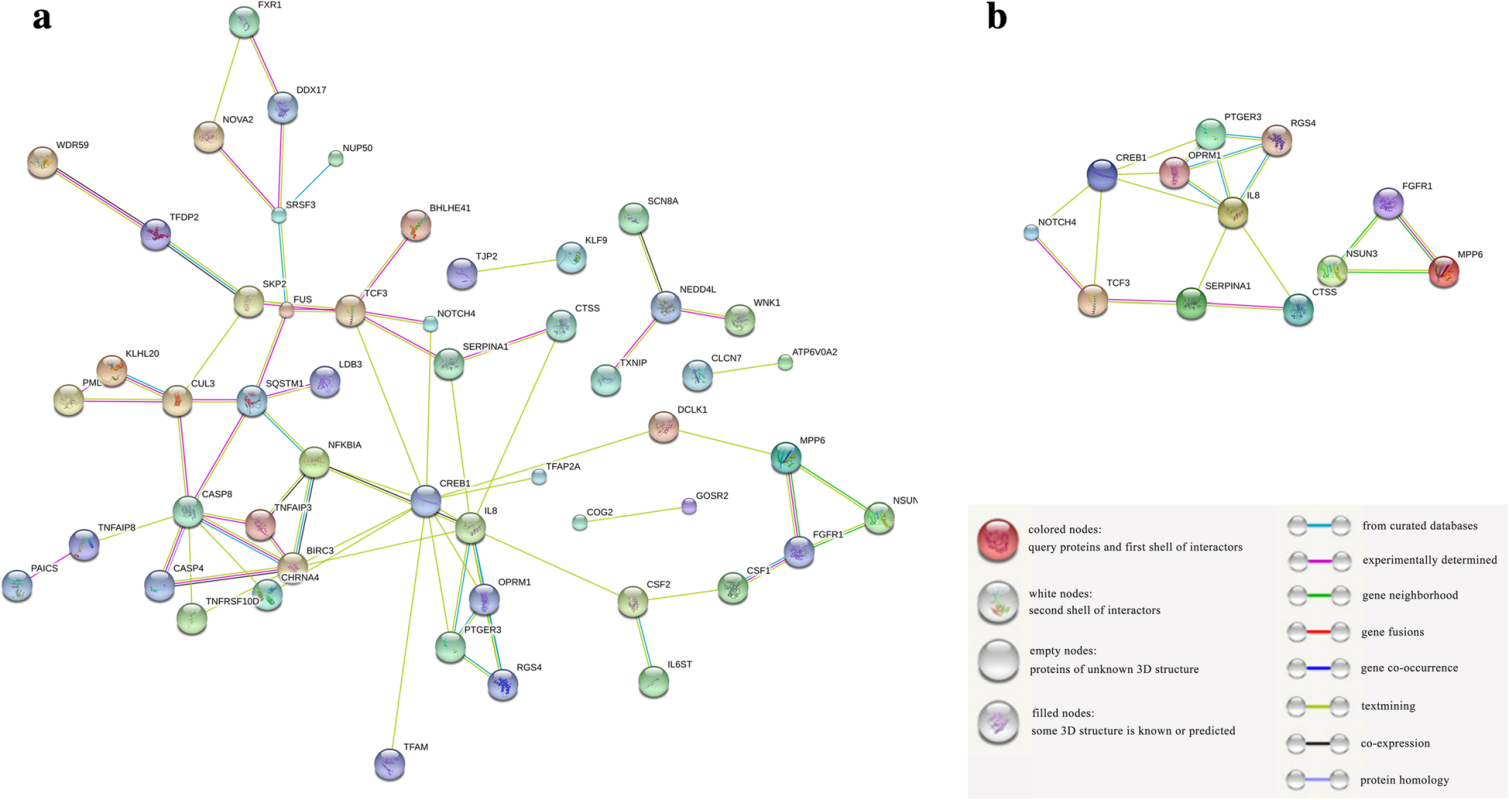
The differential expressed protein–protein interaction network and network modules. **a** Protein and protein interaction (PPI) pairs of the 116 DEGs were constructed using the STRING database, and 71 PPI pairs were derived. **b** Modules of the PPI network were analyzed using Cytoscape plugin DCOME. Three significant modules containing 12 hub genes were identified. Module 1 comprise CTSS, NOTCH4, IL8, CREB1, TCF3, and SERPINA1, module 2 comprise PTGER3, RGS4, and OPRM1, and module 3 comprise MPP6, FGFR1, and NSUN3

Next, the 116 DEGs were analyzed using the STRING database, and 71 protein-protein interactions (PPI) pairs were derived (Fig. 2a), which then underwent analysis using Cytoscape to construct PPI network. Using the plugin DCOME, we identified three significant modules consisting of the following 12 hub genes (Fig. 2b): CTSS, NOTCH4, IL8, CREB1, TCF3, SERPINA1, PTGER3, RGS4, OPRM1, MPP6, FGFR1, and NSUN3. We found using enrichment analysis that the main roles of these hub genes were in the inflammatory response.

### Verification of hub genes

**Fig. 3:**
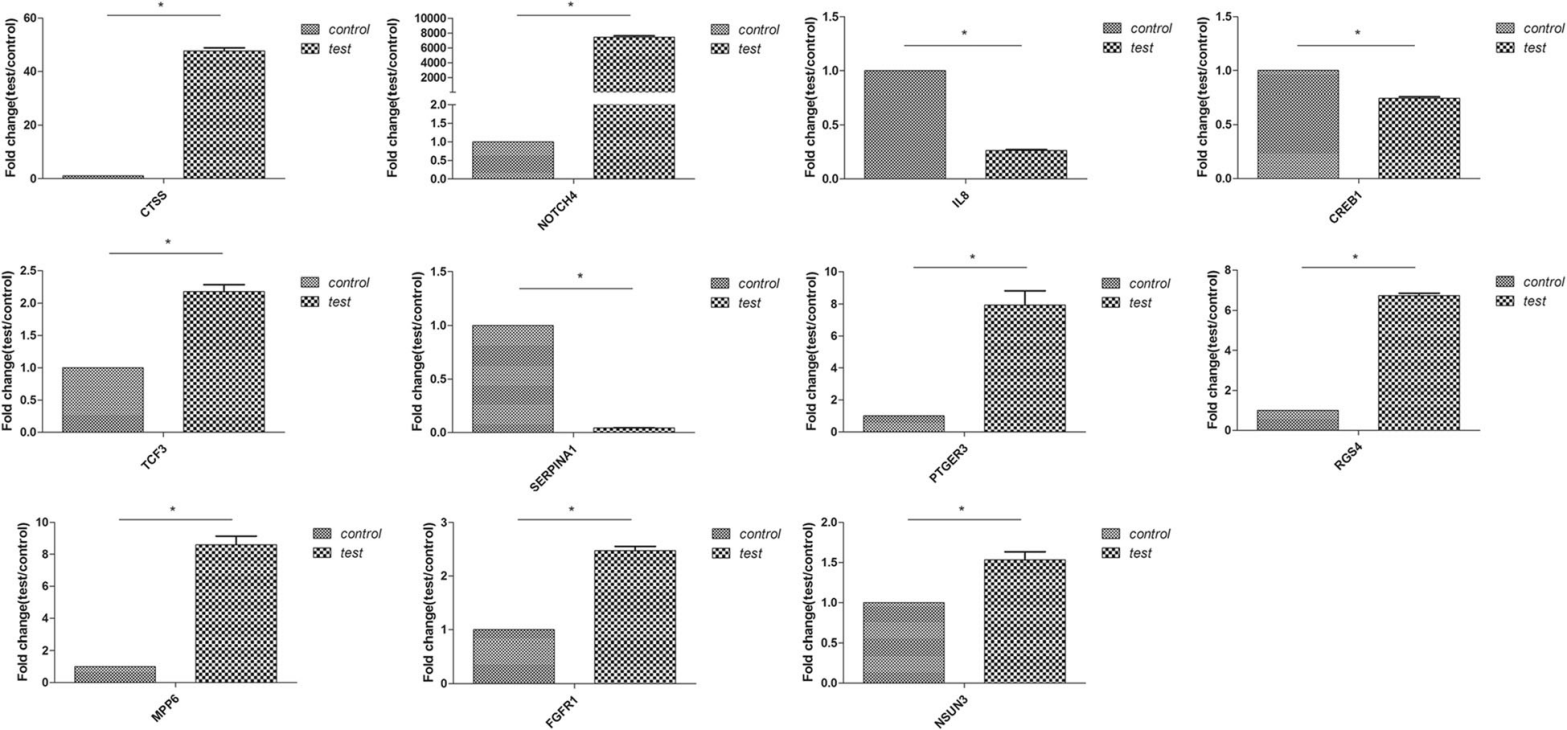
Relative expression level of hub genes in BEAS-2B cells in response to *Talaromyces marneffei*. The expression level of mRNAs was performed using qRT-PCR. Results were shown as mean ± SD, * *P* <  0.05

To verify the 12 *hub genes*, we used qRT-PCR to identify the expression level of these differentially expressed mRNAs in human bronchial epithelial cells infected with *Talaromyces marneffei* (*T.marneffei*, Fig. 3). The results of qRT-PCR showed that all hub genes, except for OPRM1 were differentially expressed (*P* <  0.05, Table 4), and were consistent with the three microarray datasets. These suggest that the 12 hub genes may function as a group, and have a role in the pathogens infection to human bronchial epithelial cells.

**Table 4:** Relative expression level of 12 hub genes in *Talaromyces marneffei* infected BEAS-2B cells

Gene	Up/down regulation	Fold change (mean ± SD)	*P*-value
CTSS	up	47.734 ± 1.16	*P* < 0.05
NOTCH4	up	7435.900 ± 186.712	*P* < 0.05
IL8	down	0.263 ± 0.009	*P* < 0.05
CREB1	down	0.746 ± 0.013	*P* < 0.05
TCF3	up	2.180 ± 0.108	*P* < 0.05
SERPINA1	down	0.044 ± 0.002	*P* < 0.05
PTGER3	up	7.941 ± 0.888	*P* < 0.05
RGS4	up	6.743 ± 0.118	*P* < 0.05
MPP6	up	8.605 ± 0.532	*P* < 0.05
FGFR1	up	2.475 ± 0.080	*P* < 0.05
NSUN3	up	1.532 ± 0.102	*P* < 0.05
OPRM1	–	–	*P>0.05*

### Text mining and prediction of miRNA

**Fig. 4:**
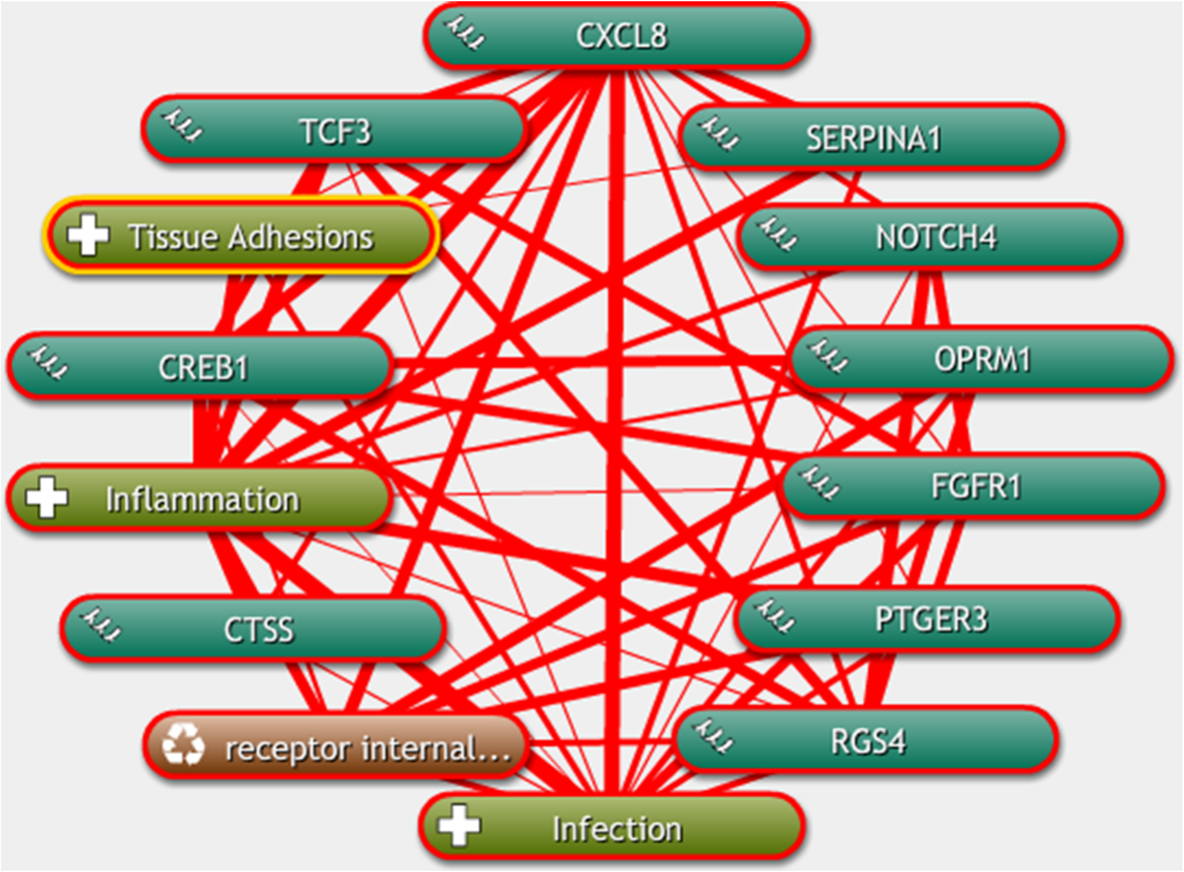
The linear relationship between the hub genes retrieved using COREMINE. Ten hub genes were associated with tissue adhesion, receptor internalization, inflammation and infection. The thicker the line, the closer the connection between the two ends

To demonstrate further the relationship between the hub genes and respiratory tract infection, text mining was performed using COREMINE. Co-occurrence analysis of the literature was conducted using “gene symbols”, “receptor internalization”, “tissue adhesion”, “inflammation”, and “infection” as search terms. We found that 10 genes (CTSS, NOTCH4, IL8, CREB1, TCF3, SERPINA1, PTGER3, RGS4, OPRM1, and FGFR1) were identified in the text-mining searches. Although all 10 of these genes were related to both inflammation and infection in the literature, we also found that two of these genes IL8 and SERPINA1 were associated with tissue adhesion, whereas six genes IL8, CREB1, RGS4, PTGER3, OPRM1, and FGFR1 were associated with and receptor internalization (Fig. 4).

Searching the miRWalk, miRanda, miRDB, RNA22, and Targetscan databases, we predicted miRNAs for the 12 hub genes previously identified. Although eight miRNAs (hsa-miR-762, hsa-miR-93-5p, hsa-miR-20a-5p, hsa-miR-3192-5p, hsa-miR-1294, hsa-miR-1972, hsa-miR-106b-5p and hsa-miR-526b-3p) were predicted by all 5 databases, we found that only one gene (TCF3) has a miRNA-target interaction. In addition, we found that hsa-miR-762 was associated with pathogen infection [8]. There was no evidence in the literature regarding the remaining miRNAs and an association with infectious diseases.

## Discussion

The GEO database includes high-throughput gene expression datasets and other functional genomics data [9]. The online tool, GEO2R is based on the R programming language and performs statistical analysis enabling users to access and analyze practically any GEO Series, regardless of data type [10]. This tool was used to analyze published microarray data in the GEO database. However, because the scope of microarray analysis in independent studies may have been limited, and that different experiments may identify different gene or mRNA targets, the robustness and reliability of these previous findings may be limited. To address this potential limitation in the present study, we used three independent microarray datasets of gene expression from airway epithelial cells responding to infection by respiratory pathogens, and collectively analyzed and evaluated all three datasets in our study. Therefore, our identification of genes that are significantly dysregulated in airway epithelial cells exposed to pathogens was identified using the collective data from three different microarray expression studies.

In the present study, we found 116 DEGs in our analysis of three independent microarray datasets. We used GO enrichment to understand better the underlying biological processes that are associated with these genes. GO is a major bioinformatics initiative to unify the characterization of gene and gene product attributes in all organisms [11]. For the present study, GO terms may identify the putative role that our set of genes may have in the process of the pathogen infection of airway epithelial cells. Our analysis of biological processes and signaling pathways demonstrated that these 116 genes were mainly involved in myeloid cell differentiation, cytokine stimulus, regulation of tyrosine phosphorylation of the STAT protein, regulation of the JAK-STAT cascade, and the NLR signaling pathway. These findings indicate that the functions of these genes are closely related to the immune response. For example, the JAK-STAT cascade is a major intracellular signaling response elicited by class I cytokine receptors, the activation of which results in direct and rapid changes in gene expression upon cytokine stimulation [12]. In addition, The NLR family pyrin domain containing 3 (NLRP3) inflammasome is a multiprotein complex that orchestrates innate immune responses to infection and cell stress through activation of caspase-1 (CASP1) and maturation of the inflammatory cytokines pro-interleukin-1β (pro-IL-1β) and pro-IL-18 [13].

To examine further the interrelationships between the DEGs, we constructed a PPI network and identified the following 12 hub genes: CTSS, NOTCH4, IL8, CREB1, TCF3, SERPINA1, PTGER3, RGS4, OPRM1, MPP6, FGFR1, and NSUN3. We found that these hub genes may function as a group and have an important role in viruses or bacteria infection. In order to investigate whether the group of hub genes is altered by particular pathogen, we further identified these hub genes by qRT-PCR in BEAS-2B cells infected by *T.marneffei*, a thermal dimorphic pathogenic fungus, which is able to cause fatal systemic infections in human. Our results showed that these hub genes involved in human bronchial epithelial cell in response to *T.marneffei.* So we posit that these genes may have clinical applications serve as biomarkers for early respiratory tract infection. In addition, we found using enrichment analysis that these hub genes were also associated with inflammation.

Text mining indicated that the majority of these hub genes were related to tissue adhesion, receptor internalization, inflammation, and infection. For example, human epithelial cells infected with Chlamydia secrete IL8, which is known to be activated by p42/44 MAPK cascade [14]. Aronoff et al. demonstrated that the prostaglandin E receptors 2 and 3 (PTGER2-PTGER3) axis has important roles in the prevention and treatment of infectious diseases [15]. In addition to the host defense response, adhesion, receptor internalization, and inflammatory responses are intrinsic to pathogen invasion. Of the 12 hub genes identified, we found that two genes were associated with tissue adhesion, six genes were associated with receptor internalization, and 10 genes were associated with inflammation. This finding demonstrates that these genes may have key roles in the process of pathogen infection in airway epithelial cells. Adhesion, receptor internalization and inflammatory response were intimately related to the invasion of pathogens and host defense against pathogens [6]. However, because there are no prior reports in the literature regarding the two hub genes MPP6 and NSUN3, and their potential role in airway epithelial cell infection, further studies to investigate their functions are warranted.

To provide additional targets for further research, we also preformed miRNA prediction of these hub genes. We found that a single gene (TCF3) has a miRNA-target interaction. Whereas among the eight candidate targets of microRNAs, hsa-miR-762 was previously reported to be associated with *Pseudomonas aeruginosa* infection [8], we did not find any prior investigations with the remaining seven in silico predicted miRNAs and infection. However, based on our findings, these miRNAs may warrant future study.

## Conclusions

In summary, by integrating the data from 3 independent microarray studies, and though an extensive functional assessment including evaluation of signaling pathways, annotation of biological processes, determination of protein-protein interactions, text mining, and identification of miRNA-target interactions, we identified a robust set of DEGs associated with airway epithelial cell response to pathogen infection. This group of genes may be tested in the sputum or bronchoalveolar lavage fluid samples as biomarkers for the diagnosis or the surveillance of early respiratory tract infections. In addition, we identified several genes and miRNAs as intriguing targets for further research. Understanding the pathogenesis will not only lead to improving prognosis and diagnosis, but also better therapeutic strategies and treatments to prevent respiratory tract infections.

## Methods

### Data collection and identification of DEGs

Three mRNA microarray expression profile datasets GSE3397 [16], GSE6802 [17] and GSE48466 [18] were retrieved from the GEO database (http://www.ncbi.nlm.nih.gov/geo/, accession numbers: GSE3397, GSE6802, and GSE48466) [9]. DEGs were screened using GEO2R, which is an interface web tool available in GEO. The identification of DEGs between human airway epithelial cells exposed to respiratory pathogens and normal cells was statistically filtered using a *P*-value < 0.05.

### Bioinformatics analysis

GO and KEGG Pathway enrichment analyses were performed using the online tool DAVID version 6.8 (https://david.ncifcrf.gov/) [19]. A *P* <  0.05 was considered statistically significant. Protein and protein interaction networks were constructed using STRING version 10.0 (http://string-db.org/) [20]. The STRING database enables a critical assessment and integration of PPI, including physical as well as functional associations [20]. DEGs were mapped into the protein search, the organism was defined as *Homo sapiens*, and the genes were queried in the interaction networks. The Molecular Complex Detection (DCOME) in Cytoscape was used to analyze the modules of the PPI network. A score greater than three was identified as hub nodes and edges. Text mining for prediction of gene function was performed using COREMINE (http://www.coremine.com/medical/) [21]. To predict miRNA-target interactions in the 3′-UTR region of genes, we used the miRWalk, miRanda, miRDB, RNA22 and Targetscan databases (http://zmf.umm.uni-heidelberg.de/apps/zmf/mirwalk2/) [22].

### *T.marneffei* strain and conidia preparation

*T.marneffei* strain was isolated from the sputum of a patient suffering from disseminated *T. marneffei* infection at the first affiliated hospital of Guangxi Medical University. *T.marneffei* was isolated as part of standard care of the patient, and was cultured on potato dextrose agar (PDA) medium (Beijing Luqiao Technology) at 25 °C for 7–10 days. Colonies were washed with sterile phosphate buffed saline (PBS), and then conidia were collected by centrifugation.

### Cell line culture and fungal infection

The human bronchial epithelial cell line BEAS-2B (ATCC® CRL-9609™) was stocked at the Experimental Center of Guangxi Medical University. Cells were cultured in RPMI1640 medium mixed with 10% fetal bovine serum (Invitrogen) at 37 °C. The test group was infected with conidia of *T.marneffei* for 4 hours.

### RNA extraction and qRT-PCR

Cells of both control and test groups were used to extract total RNA with TRIzol reagent (Invitrogen), following the manufacturer’s instructions. RNA quality and quantity were measured by Nucleic Acid Protein Detector. Total RNA was used for synthesis of cDNA with the First Strand cDNA Synthesis Kit (TaKaRa). The qRT-PCR were run using the following program: 1 cycle at 95 °C for 30 s, 40 cycles at 95 °C for 3 s, and an extension step at 60 °C for 30 s. The relative expression level of mRNAs was performed by using 2^−ΔΔ*C*T^ analysis method [23].The primers used are as follows (Table 5). GAPDH expression served as internal control.

**Table 5:** Primers used for qRT-PCR

Gene	Primer sequence (5′-3′)
CTSS-Forward	TCTCTCAGTGCCCAGAACCT
CTSS-Reverse	GTCTGAGTCGATGCCCTTGT
NOTCH4 - Forward	CTTGTCAGTCCCAACCCTGT
NOTCH4- Reverse	AGACACTCGTCCACGTCTCC
IL8- Forward	TAGCCAGGATCCACAAGTCC
IL8- Reverse	GCTTCCACATGTCCTCACAA
CREB1- Forward	GACGGAGGAGCTTGTACCAC
CREB1- Reverse	GCTGGGCTTGAACTGTCATT
TCF3 - Forward	TGCACAGCTCTCTGAAATGG
TCF3- Reverse	GGCAAAGGAGTGAAGGACAG
SERPINA1- Forward	TGCAGCCTGACTTCTTTGTG
SERPINA1- Reverse	AAGGACTCTCCTGGCCCTTA
PTGER3- Forward	GAGAGCAAGCGCAAGAAGTC
PTGER3- Reverse	CTGCTTGGACAGGTACACGA
RGS4 - Forward	ACAGGCTTAGCAGGAAGACG
RGS4- Reverse	AATTCGCAAGCAGGAAAGAA
OPRM1- Forward	ATCCCTCTTTCCTTGCCAAT
OPRM1- Reverse	GGAGTTCTCCGTCTGACAGC
MPP6- Forward	CAGCTGGTGGAAGGTCTGTT
MPP6- Reverse	TTTGTGAGTGGTCTGGTTGG
FGFR1- Forward	GAACAGGCATGCAAGTGAGA
FGFR1- Reverse	GCTGTAGCCCTGAGGACAAG
NSUN3- Forward	CTCTACATGCACGCTTTCCA
NSUN3- Reverse	GTGAAGTCGTGGGAGCAAGT
GAPDH- Forward	GCACCGTCAAGGCTGAGAAC
GAPDH- Reverse	TGGTGAAGACGCCAGTGGA

### Statistical analysis

Results are shown as mean ± standard deviation (SD) for three repeated independent experiments for each group. Statistical comparisons were conducted by using SPSS20.0 and significance was assessed by two-tailed Student’s *t*-test. Results with *p* <  0.05 were considered statistically significant.

## Additional files

Additional file 1: The GSE3397 mRNA expression profile dataset. 2033 DEGs between normal and infected airway epithelial cells were identified. (XLSX 158 kb)

Additional file 2: The GSE6802 mRNA expression profile dataset. 1241 DEGs between normal and infected airway epithelial cells were identified. (XLSX 101 kb)

Additional file 3: The GSE48466 mRNA expression profile dataset. 12950 DEGs between normal and infected airway epithelial cells were identified. (XLSX 964 kb)

## Abbreviations

DEGs: Differentially expressed genes
GEO: Gene Expression Omnibus
GO: Gene Ontology
KEGG: Kyoto Encyclopedia of Genes and Genomes
PPI: Protein-protein interactions
*T.marneffei*: *Talaromyces marneffei*
